# The impact of DNA intercalators on DNA and DNA-processing enzymes elucidated through force-dependent binding kinetics

**DOI:** 10.1038/ncomms8304

**Published:** 2015-06-18

**Authors:** Andreas S. Biebricher, Iddo Heller, Roel F. H. Roijmans, Tjalle P. Hoekstra, Erwin J. G. Peterman, Gijs J. L. Wuite

**Affiliations:** 1 Department of Physics and Astronomy, LaserLaB Amsterdam, VU University Amsterdam, De Boelelaan 1081, Amsterdam 1081HV, The Netherlands

## Abstract

DNA intercalators are widely used as fluorescent probes to visualize DNA and DNA transactions *in vivo* and *in vitro*. It is well known that they perturb DNA structure and stability, which can in turn influence DNA-processing by proteins. Here we elucidate this perturbation by combining single-dye fluorescence microscopy with force spectroscopy and measuring the kinetics of DNA intercalation by the mono- and bis-intercalating cyanine dyes SYTOX Orange, SYTOX Green, SYBR Gold, YO-PRO-1, YOYO-1 and POPO-3. We show that their DNA-binding affinity is mainly governed by a strongly tension-dependent dissociation rate. These rates can be tuned over a range of seven orders of magnitude by changing DNA tension, intercalating species and ionic strength. We show that optimizing these rates minimizes the impact of intercalators on strand separation and enzymatic activity. These new insights provide handles for the improved use of intercalators as DNA probes with minimal perturbation and maximal efficacy.

## Abstract (web summary)

DNA intercalators, a type of fluorescent probes widely used to visualize DNA, can perturb DNA structure and stability. Here, the authors show how DNA-binding affinity can be tuned using DNA tension, ionic strength and dye species, and how this can be used to minimize DNA structural perturbations.

## Graphical abstract

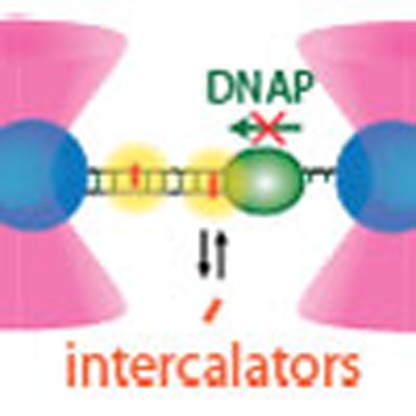

DNA intercalators are small molecules that can reversibly bind in between adjacent basepairs of double-stranded DNA (dsDNA)^1^. Cyanine intercalating dyes such as YOYO-1 exhibit a large enhancement of fluorescence on intercalation^2^, which makes them very attractive probes to report on the presence, location and spatial extent of dsDNA in single-molecule and bulk assays^3^,^4^. Similarly, enzymatic reactions on DNA have been interrogated by monitoring, for example, the displacement of fluorescent intercalators by helicases^5^,^6^ or the binding of intercalators to DNA that is newly synthesized by polymerases^7^. Intercalation is also known to play a more malignant role in disease and drug treatment, where it is known to disturb essential DNA-associated processes such as replication, transcription and repair^8^,^9^,^10^,^11^.

For intercalating dyes it is well known that they alter the structure and mechanical properties of DNA^1^,^12^,^13^. In turn, these structural alterations can perturb enzymatic reactions on DNA^8^,^9^,^10^. Whereas such perturbation is highly undesirable in biochemical assays, fatal perturbation is the desired function of intercalator drugs^8^. Such perturbations are enhanced when the intercalator's affinity for DNA is higher^10^. To understand intercalator-induced perturbations, a thorough knowledge of the mechanisms and kinetics of intercalation is required. Ultimately, this knowledge could be exploited to control adverse effects of intercalators and optimize intercalator-based experimentation and treatment.

Several studies have addressed DNA–intercalator interactions^12^,^13^,^14^,^15^,^16^,^17^,^18^. It has been established that DNA intercalation by cyanine dyes lengthens DNA (that is, increases its contour length)^13^,^17^, while the bending rigidity (that is, the persistence length) of DNA is not affected^15^,^16^. Structural studies indicate that the DNA lengthens by 0.34 nm for each intercalated moiety^19^,^20^,^21^,^22^,^23^ and that the footprint is two basepairs per moiety because of neighbour exclusion^24^. Less is known about the binding kinetics of DNA-intercalating dyes. Their affinity for DNA was shown to depend on DNA tension, and equilibration rates were found to range from a fraction of a second up to hours^12^,^14^,^15^,^25^. Lacking are, however, a complete understanding of the binding mechanism, a characterization of the association and dissociation rates and their dependence on DNA tension^14^,^15^.

Here we thoroughly investigated intercalation of DNA by four mono-intercalating cyanine dyes YO-PRO-1 (YO), SYTOX Orange (SxO), SYBR Gold (SbG) and SYTOX Green (SxG), and two bis-intercalating dyes YOYO-1 (YOYO) and POPO-3 (POPO), as function of DNA tension and ionic strength. To this end, we combined force-extension analysis using optical tweezers with fluorescence microscopy capable of visualizing individual DNA-bound intercalators^26^,^27^,^28^,^29^. This correlative approach provided access to a much wider range of kinetic rates and affinities than previously accessible with the individual techniques. We show that all results are fully consistent with a single-step binding mechanism. Moreover, we demonstrate that, with judicious selection of intercalator, buffer and tension, the intercalator-based interrogation of processes such as DNA polymerase activity and DNA melting can be significantly optimized.

## Results

**Figure 1:**
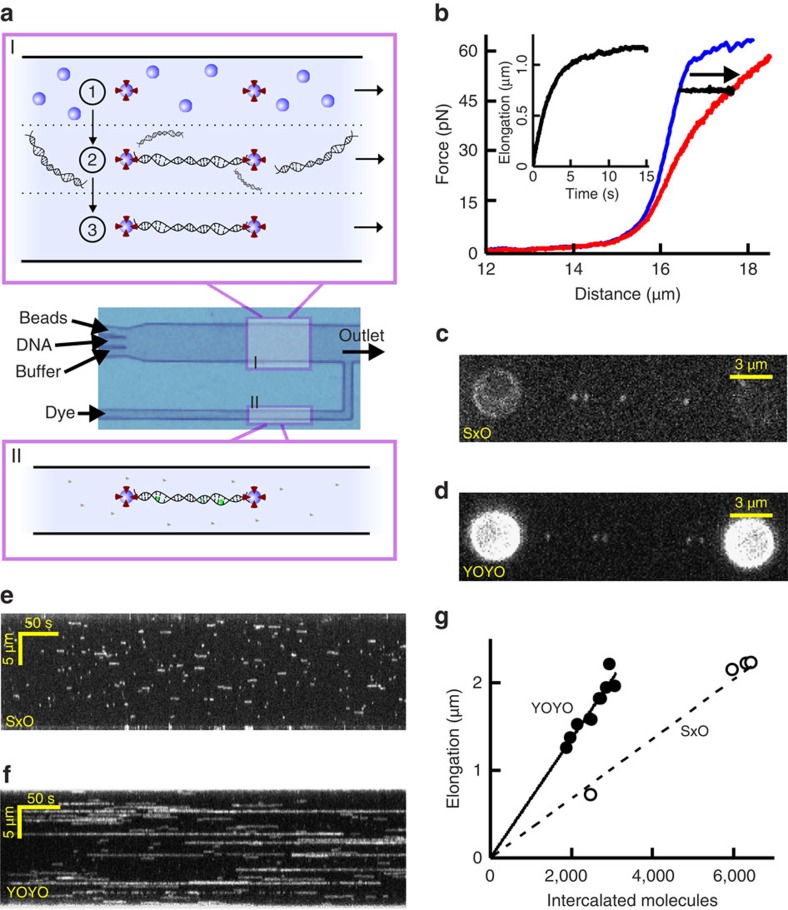
Experimental approach. (**a**) Multichannel laminar flow cell used for (I), optical trapping of two microspheres (1), tethering of DNA between the microspheres (2) and mechanical characterization of the tethered DNA (3) and for (II), immersing the DNA dumbbell in an intercalator-containing buffer by moving the flow cell with respect to the optical traps using stage motion. (**b**) Force-extension curves of bare DNA (blue) and DNA with an equilibrated coverage of SxO (red). Black curve and inset: equilibration dynamics monitored by determining DNA elongation going from bare DNA to intercalated DNA at a constant tension of 48 pN. (**c**,**d**) Fluorescence microscopy images of individual DNA-bound SxO (**c**) and YOYO (**d**) dyes. Scale bars, 3 μm. (**e**,**f**) Kymographs show real-time binding and dissociation dynamics of individual SxO (**e**) and YOYO (**f**) molecules. Scale bars, 50 s (horizontal), 5 μm (vertical). (**g**) DNA elongation (measured with optical tweezers) as a function of the number of bound intercalators (obtained from fluorescence intensity as detailed in Methods). A linear fit yields the length increase per dye (Δ*x*_eq_), 0.68±0.04 nm (solid) and 0.34±0.03 nm per dye (dashed line).

In our experiments, we manipulate single bacteriophage lambda DNA molecules using optical tweezers^26^,^29^: two streptavidin-coated microspheres are optically trapped and tethered to the biotinylated ends of a dsDNA molecule inside a microfluidic chamber (Fig. 1a)^30^. The tethered DNA can be moved to different channels of our multichannel laminar flow cell, which allows rapid and complete buffer exchange (Fig. 1a, Methods). The capability to switch channels for buffer exchange is particularly important for cyanine intercalators, which are well known to adsorb to glass surfaces and consequently cause buffer contamination by slow desorption^25^,^31^. We monitor the dye coverage of the DNA in two complementary ways (Methods). First, we measure the changes in DNA elongation, Δ*L*, that result from intercalator (un)binding using optical tweezers (Fig. 1b, Δ*L* is calculated as the end-to-end-length difference between intercalated and bare DNA)^12^. This step assumes that Δ*L* at a given dye coverage is independent of tension (see discussion below). Second, we employ wide-field fluorescence microscopy to quantify the fluorescence intensity increase of DNA-bound dyes. The sensitivity of our fluorescence set-up is high enough to observe individual molecules of the photostable dyes SxO and YOYO binding to the DNA (*cf.* discrete events in Fig. 1c,d, Supplementary Fig. 1). Kymographs obtained from a time series of images taken under such conditions directly reveal that SxO intercalation events are much shorter than those of YOYO, under identical conditions (Fig. 1e,f). By correlating single-dye fluorescence data to elongation data (Fig. 1g), we can directly measure the equilibrium DNA elongation per intercalator, Δ*x*_eq_ (Methods), yielding 0.34±0.03 nm per mono-intercalator and 0.68±0.04 nm per bis-intercalator, within error of values based on structural data^19^,^20^,^21^,^22^,^23^.

### Tension dependence of DNA intercalation affinity

**Figure 2:**
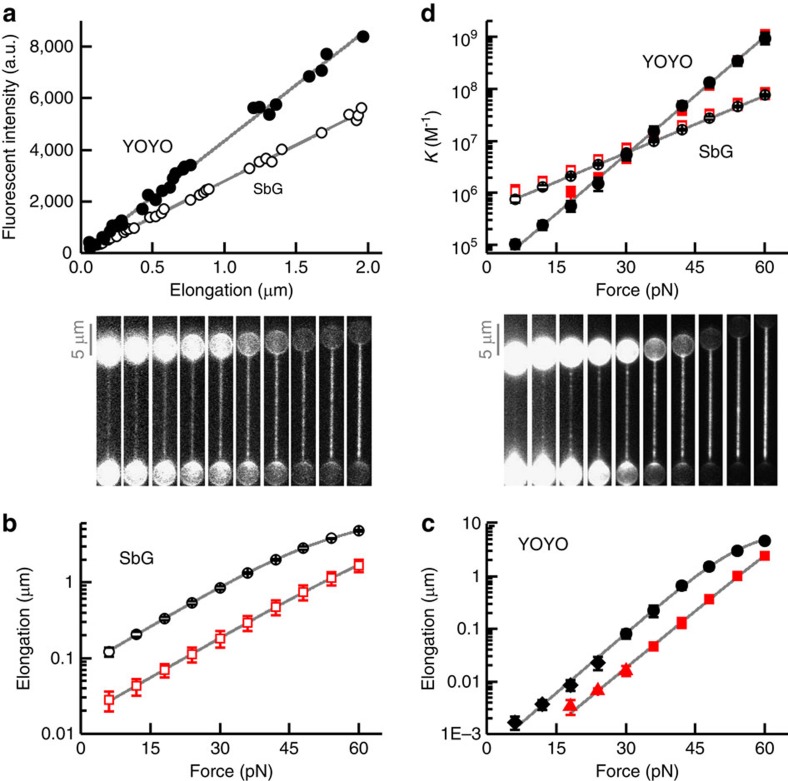
Force-dependent DNA intercalation of a mono- (SbG, open symbols) and a bis-intercalator (YOYO, solid symbols) at 1,000 mM NaCl. (**a**) Total fluorescence intensity as a function of DNA elongation. Solid lines: linear fits through origin. (**b**,**c**) DNA elongation and representative fluorescence images (normalized intensity) as a function of tension. Solid curves: fits to data (equation (5) and Methods). The DNA tension for each fluorescence image corresponds to the force axis below. Scale bars, 5 μm. (**d**) Binding constant as a function of tension, calculated using elongation data of **b**,**c**. Solid lines: exponential fits. (**a**–**d**) Open red squares: 1.6 nM SbG; open black circles, 10 nM SbG, filled red squares: 0.1 nM YOYO; filled black circles: 0.45 nM YOYO; triangles/diamonds: DNA elongation calculated from total fluorescence intensity. Error bars s.e.m., *n*≥5.

**Figure 3:**
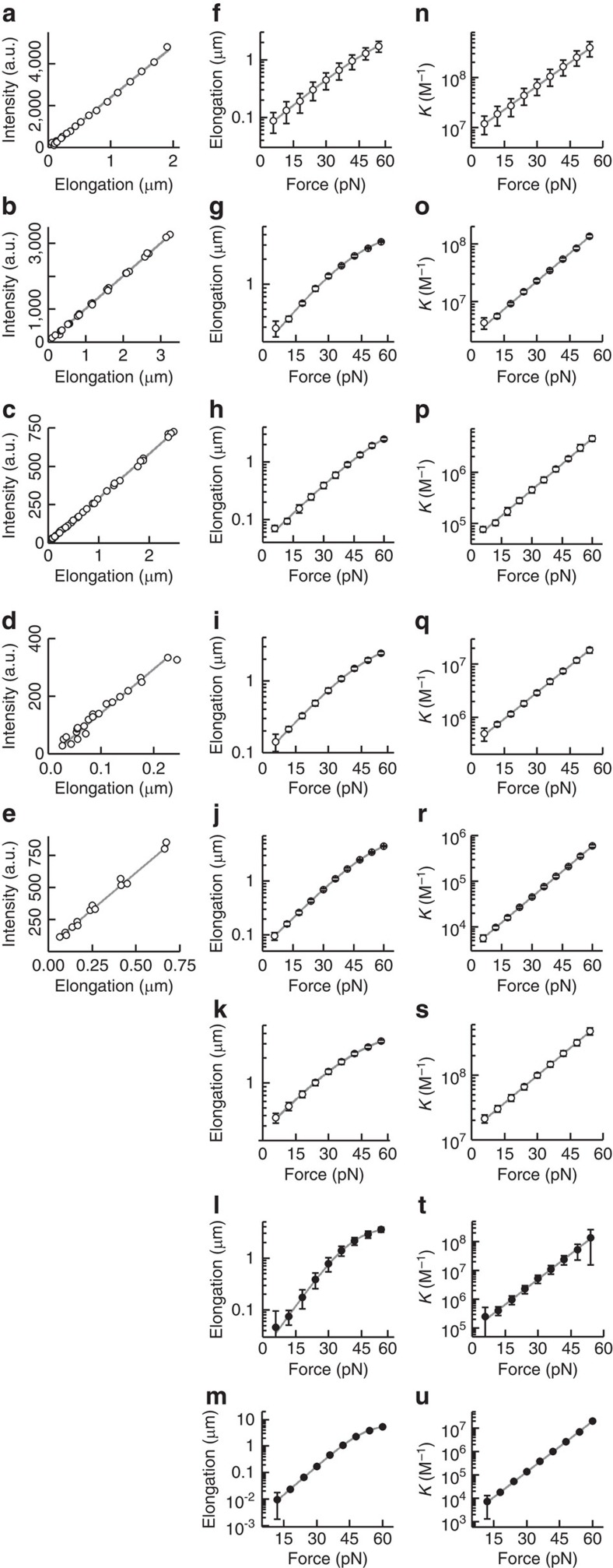
Force-dependent equilibrium binding data of mono- (open symbols) and bis-intercalators (closed symbols). In all cases, the fluorescence increase scales linearly with elongation (**a**–**e**), the elongation versus force can be well fitted with the multiligand binding model (**f**–**m**) and the resulting affinity constant *K* scales single-exponentially with force (**n**–**u**). Grey curves are linear fits (**a**–**e**), fits of equation (5) (Methods, **f**–**m**) and fits of equation (2) (**n**–**u**). (**a**,**f**,**n**) Overall, 0.5 nM SbG with 100 mM NaCl; (**b**,**g**,**o**) 5 nM SxO with 100 mM NaCl; (**c**,**h**,**p**) 20 nM SxO with 1,000 mM NaCl; (**d**,**i**,**q**) 20 nM YO with 100 mM NaCl; (**e**,**j**,**r**) 1.0 μM YO with 1,000 mM NaCl; (**k**,**s**) 1.5 nM SxG with 100 mM NaCl; (**l**,**t**) 7 nM POPO with 100 mM NaCl; (**m**,**u**) 40 nM POPO with 1,000 mM NaCl. For SxG and POPO, the scaling of elongation with fluorescence could not be reliably performed because of the low signal-to-noise ratios and the high photobleaching rates. Error bars, s.e.m., *n*≥5.

In a first set of experiments we quantified the DNA-binding affinity of the intercalators as a function of tension by monitoring the equilibrated fluorescence intensity and the DNA elongation in response to changes in DNA tension (Figs 2 and 3). We first verified that the elongation scales linearly with the total fluorescence intensity (Figs 2a and 3a–e), which confirms that both signals are directly proportional to dye coverage. The equilibrium coverage increases exponentially with tension and levels off slightly, at higher elongation (Δ*L*>1 μm), due to saturation effects (Figs 2b,c and 3f–m). All mono-intercalators studied exhibited an increase in dye coverage of about two orders of magnitude over the measured force range of 6–60 pN (Fig. 2b, SbG (1,000 mM NaCl); Fig. 3f–k, SbG (100 mM NaCl), SxO, YO and SxG). Strikingly, the bis-intercalators YOYO and POPO exhibited an even stronger increase in coverage (four orders of magnitude) over the same tension range (Fig. 2c, YOYO; Fig. 3l,m, POPO). Note that our sensitive fluorescence-force approach enables quantification of very low dye coverages (elongation <2 nm in Fig. 2c, given the size of lambda, this corresponds to less than 1 dye per 10,000 bp), which allows accurate quantitative analysis of dye coverage over a range that spans nearly four orders of magnitude.

To extract the tension-dependent DNA-binding constant *K*(*F*) from elongation data, we use a fitting procedure based on the following multiligand binding isotherm (Methods and Supplementary Note 1):


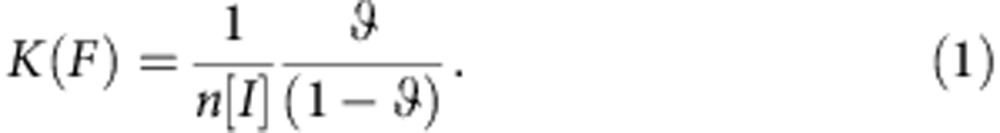


Here *n* is the (apparent) footprint in basepairs, [*I*] is the intercalator concentration and 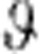 is the fractional dye coverage (0≤
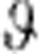
≤1, with 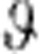
=1 corresponding to full coverage). It has been validated before that, for intercalators, *K* exhibits a single-exponential force dependence because the external stretching force reduces the net free energy of intercalation^12^:


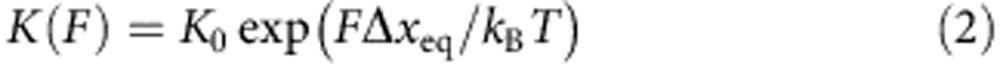


Here the ratio *k*_B_*T*/Δ*x*_eq_ represents a characteristic force, *Φ*_eq_, a measure of the tension dependence of *K*(*F*) (*K* depends on tension with a factor exp(*F*/*Φ*_eq_))^11^. Fits based on equations (1) and (2) (equation (5) as described in Methods) indeed reveal that *K*(*F*) increases single exponentially with tension (*cf.* Figs 2d and 3n–u, which was calculated from the fits and data in Figs 2b,c and 3f–m, respectively) and that *K* is independent of dye concentration, as expected. The fits also yield the DNA-binding constant at zero tension, *K*_0_ (Table 1). Furthermore, analysis of our data using the multiligand binding isotherm is in good quantitative agreement with a separate analysis using the more complex, noncooperative McGhee-von Hippel binding isotherm (Supplementary Note 1, Supplementary Fig. 2 and Supplementary Table 1)^32^.

For the mono-intercalator studies, we obtained characteristic forces of ∼12 pN (Fig. 2b,d), corresponding to an equilibrium elongation (Δ*x*_eq_) that is in good agreement with the expected value of 0.34 nm (Table 1)^2^,^22^. For the investigated bis-intercalators (Fig. 2c,d, Table 1), characteristic forces of ∼6 pN were obtained. This stronger force dependence agrees well with the equilibrium extension of a bis-intercalator being twice that of a mono-intercalator (*cf.* equation (2)).

### Tension-dependent kinetics of DNA intercalation

**Figure 4:**
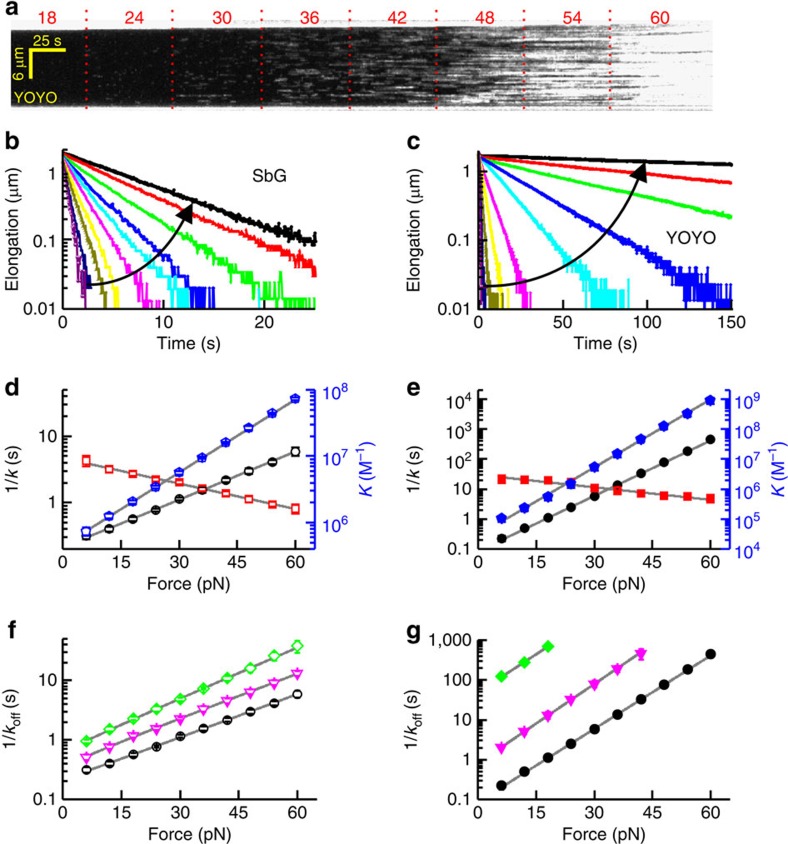
Intercalation kinetics of bis-intercalator YOYO (**a**,**c**,**e**,**g**) and mono-intercalator SbG (**b**,**d**,**f**). (**a**) Kymograph of YOYO-binding events, acquired at a tension that increases from 18 to 60 pN (indicated in pN above the kymograph). The average event duration can be clearly seen to increase with tension. Scale bar, 25 s (horizontal), 6 μm (vertical). (**b**,**c**) De-intercalation of the dye in intercalator-free buffer can be followed by the decrease in DNA elongation over time at constant tension. The displayed decay curves are measurements at different tension at 1,000 mM NaCl (increasing from 6 to 60 pN in 6 pN increments, in the direction indicated by the arrow). (**d**,**e**) Force dependence of 1/*k*_off_ (black circles), *K* (blue pentagons) and 1/[*I*]*k*_on_ (red squares) for SbG (**d**) and YOYO (**e**) at 1,000 mM NaCl. (**f**,**g**) Force dependence of 1/*k*_off_(*F*) at varying [NaCl]. Green diamonds, 100 mM NaCl; magenta triangles, 300 mM NaCl; black circles, 1,000 mM NaCl. Solid grey lines represent single-exponential fits to the data. Error bars, s.e.m., *n*≥5 (**d**–**g**).

**Figure 5:**
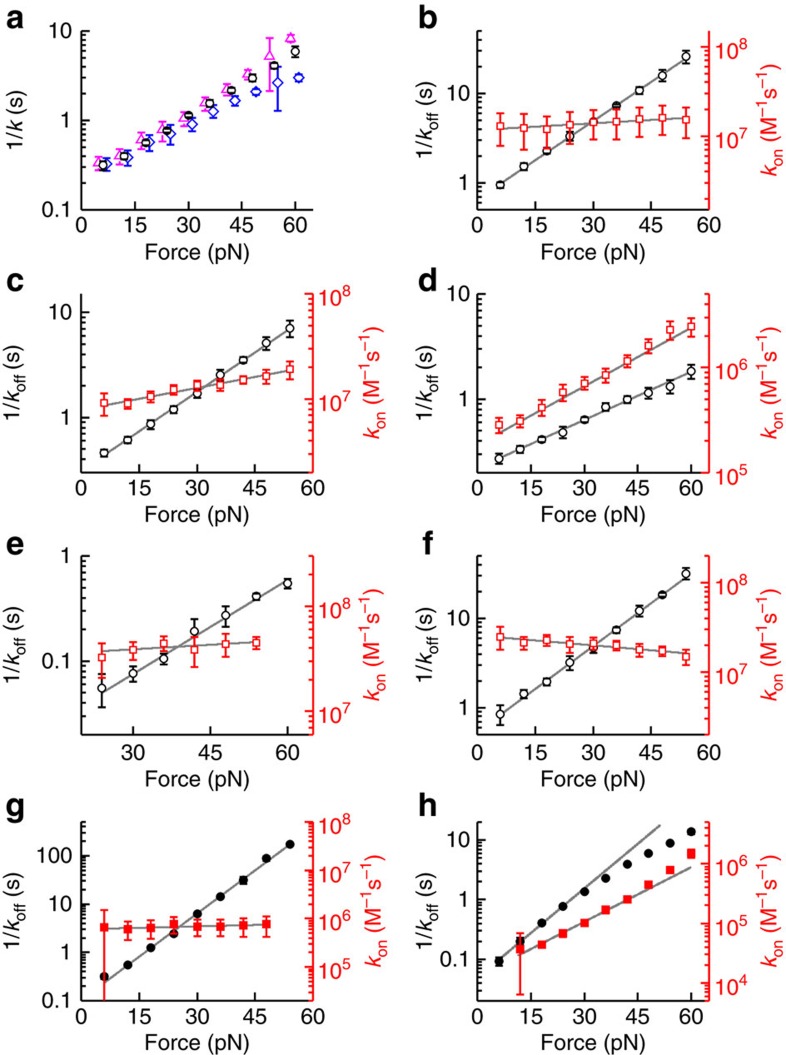
Kinetics of dye-binding for mono- (open symbols) and bis-intercalators (closed symbols). (**a**) Comparison of measured *k*_off_ values (black circles) with the corresponding equilibration rate *k*_eq_ (blue diamonds) for 10 nM SbG at 1,000 mM NaCl reveals that the difference between *k*_eq_ and *k*_off_ scales with the dye coverage (*cf.* equation (6), Methods) such that significant deviations are only observed at high tension. A direct calculation of *k*_off_ based on the measured 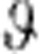 and *k*_eq_ and equation (6) (blue diamonds) shows that the measured and calculated *k*_off_ are in good agreement. (**b**–**h**) Measured (inverse) off- (black circles) and on-rates (red squares). SbG with 100 mM NaCl (**b**); SxO with 100 mM NaCl (**c**) and 1,000 mM NaCl (**d**); YO (**e**) and SxG (**f**) with 100 mM NaCl; POPO with 100 mM NaCl (**g**) and 1,000 mM NaCl (**h**). Grey curves show exponential fits of the data. The fits in **h** are restricted to the force range 0<*F*≤24 pN because *k*_off_(*F*) exhibited a nonexponential force dependence at higher forces. Error bars, s.e.m., *n*≥5.

In order to elucidate the kinetics of DNA intercalation, we investigated whether the association rate (*k*_on_) or dissociation rate (*k*_off_) constituting the binding constant (*K*=*k*_on_/*k*_off_) are affected by tension. Single-dye fluorescence microscopy shows that the duration of individual DNA-binding events (which is on average 1/*k*_off_) increases strongly with tension (Fig. 4a), which implies that *k*_off_ is tension-dependent. To directly obtain *k*_off_ we quantified the detachment of intercalators after rapidly moving intercalated DNA into a microfluidic channel lacking intercalator in solution (Methods). We ensured that the off-rates thus measured are the result of complete de-intercalation, that is, the extension in the end matches that of uncoated DNA (Fig. 4b,c and Supplementary Fig. 3). In cases where the off-rate is too fast for channel exchange, *k*_off_ could also be estimated from the equilibration rate (*k*_eq_, *cf.* Supplementary Note 1 and Fig. 5a) in the presence of intercalator^10^. In the dissociation experiments, we observed that the extension at constant tension decreased single exponentially over time, which indicates the presence of a single, rate-limiting step (Fig. 4b,c). Performing these experiments at different tensions yielded dissociation rates that decrease exponentially with increasing tension (*cf.* 1/*k*_off_ in Figs 4d,e and 5b–h). By fitting *k*_off_(*F*)=*k*_off,0_ × exp(*F*/*Φ*_off_), the characteristic forces of the dissociation rates (*Φ*_off_) can be obtained (Table 1). We, furthermore, obtain the association rate using the relation *k*_on_(*F*)=*k*_off_ × *K*=*k*_on,0_ × exp(*F*/*Φ*_on_), on corresponding data sets for *K*(*F*) and *k*_off_(*F*) (Figs 4d,e and 5b–h, Table 1). We observed that the tension dependence of the association rates was generally much weaker than that of the corresponding dissociation rate. This shows that the strong increase in the equilibrium binding constants of these intercalators with DNA tension is mostly caused by a strong decrease in the dissociation rate. From these findings, it also follows that the off-rate of bis-intercalators is intrinsically much more force-dependent than that of mono-intercalators because of the twofold longer distance to the transition state for bis-intercalators than for mono-intercalators, which makes the change in energy barrier height on applying force larger (see Discussion).

In previous studies it was observed that the binding kinetics of YOYO depend strongly on salt concentration^15^,^33^. To unravel the kinetic origin of this effect we determined the dissociation rates at various concentrations of monovalent salt (Fig. 4f,g). The single-exponential data fits showed that changing the ionic strength mostly alters the zero-force dissociation rate, rather than the characteristic force (Table 1). For YOYO we find a stronger salt dependence than for the mono-intercalator SbG. This is probably because bis-intercalators contain more charges than the mono-intercalators, emphasizing the important role of the electrostatic interaction of positive residues of the cyanine dyes with the negatively charged phosphate backbone of the DNA^34^.

Our investigations have thus far yielded a quantitative characterization of DNA intercalation kinetics. In particular, we reveal that the off-rates can be tuned over seven orders of magnitude by altering DNA tension, changing the intercalating species and varying the ionic strength. Such experimental control of reaction kinetics can be used to elucidate the influence of intercalators and their kinetics on DNA and DNA processing.

### Impact of intercalator binding on DNA mechanics

**Figure 6:**
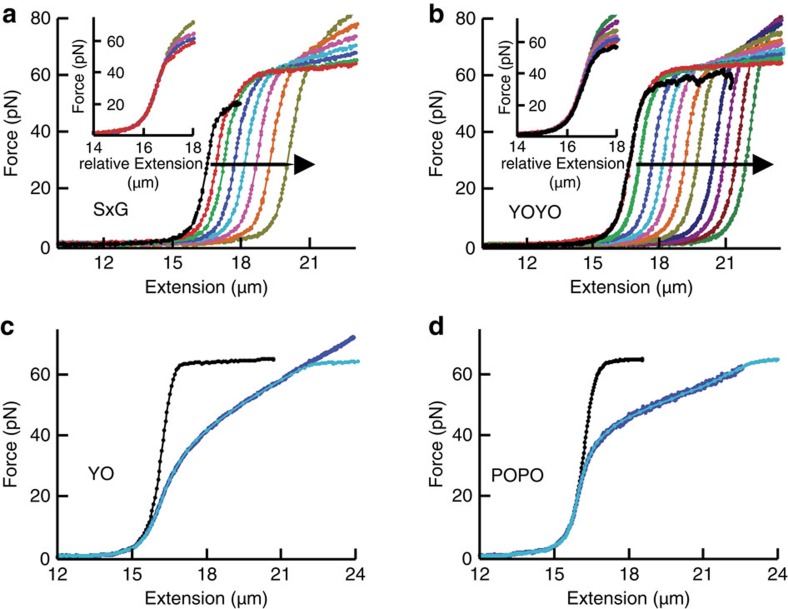
Impact of intercalators on DNA mechanics. (**a**,**b**) Nonequilibrium force-extension curves of SxG (**a**) and YOYO (**b**) at 10 and 25 mM NaCl, respectively, at increasing dye coverage (direction of increase indicated by arrows). For each curve, the dye coverage is essentially constant (Methods). Insets, curves shifted horizontally to overlay low-force regions. (**c**,**d**) Equilibrium force-extension curves in absence (black) and presence (blue) of YO (**c**) and POPO (**d**) at 1,000 mM NaCl. Cyan, fits to data compiled from the black curve using equation (4).

To investigate the mechanical impact of intercalators on DNA, we first selected conditions that result in very slow intercalation kinetics such that force-extension curves of DNA can be obtained at effectively constant dye coverage. These conditions facilitate an unambiguous mechanical analysis of the intercalated DNA (Methods) and are satisfied for the mono- and bis-intercalators with the lowest off-rates (SxG and YOYO) at low ionic strength (Fig. 6a,b). An overlay of the shifted curves, acquired at different constant dye coverages, revealed that DNA intercalation merely changes the DNA contour length (Fig. 6a,b, insets), and that neither persistence length^15^,^16^ nor extensibility were affected (Supplementary Fig. 4a,b). Several lines of evidence indicate that this finding is valid for all the cyanine intercalators studied here (Supplementary Note 2). The mechanical impact of these cyanine intercalators on the DNA end-to-end length can thus be described by a linear shift to longer extension, that is,


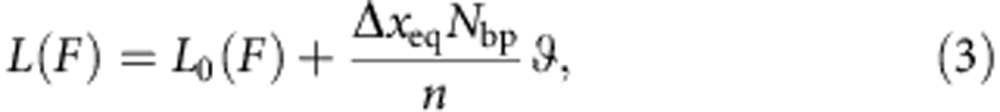


where *L*_0_(*F*) is the DNA extension (end-to-end length) without intercalation and *N*_bp_ is the number of basepairs in lambda DNA (*N*_bp_=48,502), such that 
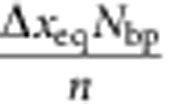
 denotes the maximum DNA elongation at full coverage (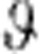
=1, see also Methods). This finding justifies that, for the studied cyanine intercalators, 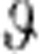 can be obtained from the relative elongation at forces below the overstretching regime and thus validates the above binding analysis using equation (5).

Binding conditions that induce fast intercalation provide access to an opposite kinetic regime, where DNA intercalation is always in equilibrium and the equilibrated dye coverage varies with tension. Under these conditions, force-extension curves deviate substantially from those obtained without intercalators (Fig. 6c,d). We can use equation (3) to also describe the equilibrium regime if we substitute 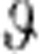 by the force-dependent equilibrium dye coverage obtained from equations (1) and (2), such that:


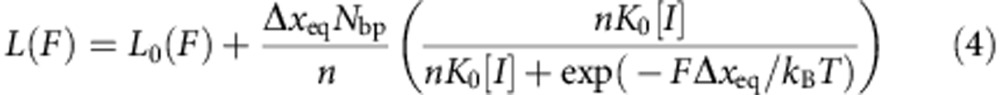


Indeed, equation (4) provides a remarkably good quantitative description of the equilibrium-intercalated force-extension curves, providing further evidence that equation (3) is valid also for the dyes used in these experiments, YO and POPO (*cf.* Fig. 6c,d). In addition, fitting force-extension curves using equation (4) allows estimating parameters *K*_0_, *n* and Δ*x*_eq_ from a single measurement.

### Intercalator impact on DNA melting and enzymatic activity

**Figure 7:**
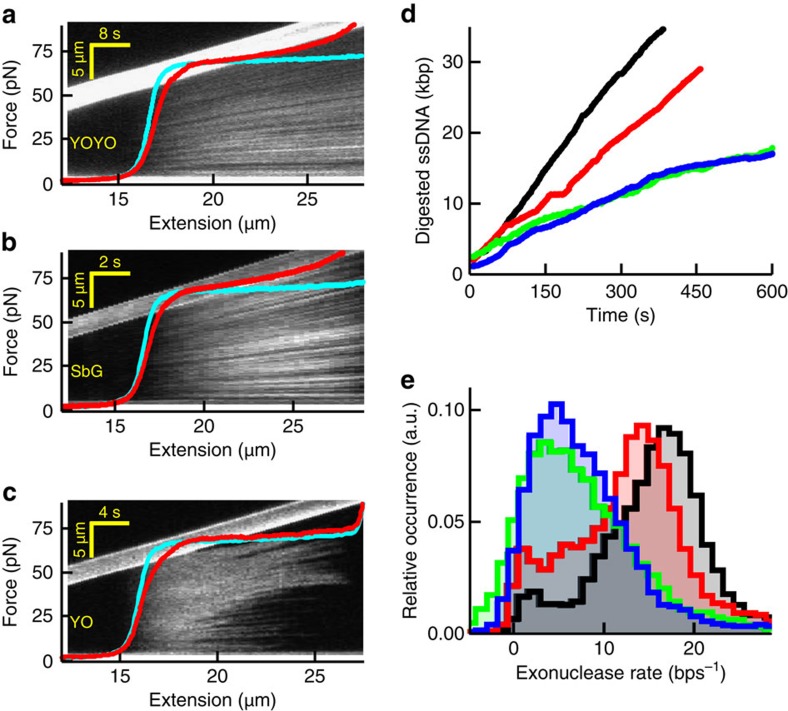
Impact of intercalators on DNA overstretching and DNA-enzyme activity. (**a**–**c**) Kymographs and corresponding force-extension curves (red) in the presence of YOYO (**a**), SbG (**b**) and YO (**c**) at 100 mM NaCl. The kymograph image and corresponding force-extension curves are co-aligned along the horizontal axis, which is possible because of the constant stretching speed of the moving bead (visible as the bright, upward tilted bar in the image). Thus, the fluorescence pattern in the vertical direction in the kymograph is a DNA-staining pattern at the DNA extension indicated on the horizontal axis. Cyan curves, no intercalator present. (**d**) Time traces recorded at 40 pN showing dsDNA digestion by the exonuclease activity of T7 DNA polymerase. Black, no intercalator; red, YO; blue, SxO; green, SbG. Under these experimental conditions, YOYO abolished all exonuclease activities. (**e**) Histograms of the resulting average digestion rates obtained at the four different conditions (Methods).

Finally, we set out to investigate how intercalators and their kinetics have an impact on DNA overstretching and enzymatic processing of DNA. DNA overstretching is a cooperative transition during which the two complementary DNA strands can unwind and—depending on the salt conditions—separate (melt)^35^. It is characterized by a wide plateau in the force-extension curve at ∼65 pN (Fig. 7a). In the presence of the bis-intercalator YOYO, however, the force plateau was replaced by a monotonic force increase, demonstrating that the cooperativity of the transition was perturbed (Fig. 7a, red curve). The corresponding kymograph obtained from concomitantly measured fluorescence images showed that intercalator binding and DNA elongation occur homogeneously over the DNA molecule. These two observations suggest that intercalated DNA is stabilized and protected against unwinding and melting, and that overstretching cannot trigger intercalator unbinding. Similar results were obtained for the mono-intercalator SbG (Fig. 7b), indicating that these effects are not specific for bis-intercalators. Markedly different results were obtained in DNA-overstretching experiments with YO present in solution, which has a much faster dissociation rate than both YOYO and SbG: the kymograph in Fig. 7c shows that the binding pattern is relatively homogeneous at low extensions, converting to a heterogeneous pattern exhibiting extended ‘dark' regions at extensions above 18 μm. The force-extension curve, on the other hand, exhibits a relatively unperturbed force plateau. Both observations suggest that, despite YO being present in solution, the intercalated DNA could still overstretch in a cooperative manner and that the intercalator unbinds during the process of DNA unwinding and melting^35^. We investigated DNA intercalation under various conditions (Supplementary Fig. 5) and concluded that these differences in overstretching characteristics are explained by the differences in intercalator kinetics: an intercalator that exhibits an off-rate that is fast compared with the timescale of stretching (for example, YO in Fig. 7c) unbinds fast enough to not disturb the cooperative overstretching transition.

Finally, we tested whether this minimal perturbation by intercalators with fast off-rates also applies to DNA processing by enzymes. To this end we studied the exonuclease activity of T7 DNA polymerase (DNAP)^36^ on a DNA template containing a single 3′-recessive end^37^. We chose conditions under which fast and processive DNAP exonuclease activity can be observed, and monitored the reduction of enzymatic activity in the presence of various mono-intercalators (Fig. 7d, Methods). Exonuclease rates obtained from such measurements demonstrated that the smallest reduction in activity is found for YO, the intercalator with the fastest unbinding kinetics (Fig. 7e), in line with the DNA-overstretching experiments above. We also found that the polymerase activity of the same enzyme is not appreciably affected by YO under identical conditions (Supplementary Fig. 6). These findings reveal that the application of intercalators with high DNA-unbinding rates minimizes the effect on *in vitro* enzyme activity.

## Discussion

In this study, we have shown that DNA binding by mono- and bis-intercalating cyanine dyes obeys single-step binding kinetics that depend strongly on DNA tension. Our analysis yielded remarkably consistent results for the six intercalators investigated, suggesting that our findings are valid for mono- and bis-intercalating cyanine dyes in general.

Intriguingly, our investigation revealed that the strong force dependence of the binding constant is mainly governed by the off-rate, whereas the on-rate is far less sensitive to force. This difference in force dependence provides information about the transition state: if a process requires a large DNA length change in order to reach the transition state, then its reaction rate will be highly sensitive to an externally applied force. The larger force dependence of the off-rate thus implies that the transition state lies further away from the intercalated state (on average, Δ*x*_off_ is 0.25 nm for mono- and 0.55 nm for bis-intercalators, *cf.* Table 1) than from the non-intercalated state (Δ*x*_on_ is 0.06 nm for mono- and 0.15 nm for bis-intercalators). This finding can, in fact, be rationalized by the Hammond–Leffler postulate, which states that exothermic reactions have a transition state close to the educts^38^,^39^. Indeed, most intercalation reactions have been shown to be exothermic^40^,^41^,^42^. It could be interesting to further study the general applicability of this postulate to DNA binding by, for example, proteins.

It is remarkable that DNA intercalation can be completely understood in terms of a straightforward, first-order, single-step binding process. Specifically, our results indicate that the actual intercalation is rate-limiting and all preceding processes occur on timescales below the resolution of our instrument (<0.1 s). Previously, more complex binding models had to be invoked in order to describe intercalation equilibrium and kinetics^14^. We believe that saturation and out-of-equilibrium effects (see above) that had not been accounted for consistently might explain the discrepancy with our findings. It has also been proposed that some intercalators display a stable secondary binding mode from which, in the absence of dyes in solution, intercalation can occur^15^. This additional binding mode was interpreted to be non-intercalating DNA-backbone binding. We did not find evidence for this interpretation. In our hands, such an effect was only observed under conditions that intercalators can desorb from the glass surface.

The kinetics of DNA intercalation are, under most conditions, independent of dye coverage. At monovalent salt concentration up to 300 mM, however, we observe an elevated off-rate at high dye coverage (Supplementary Fig. 7), resulting in deviations from single-exponential kinetics. We attribute this to electrostatic repulsion of the positively charged dyes, which can be suppressed by the increased screening at high salt concentration. Such repulsion has been suggested before^43^ and can also explain the observed increase in the apparent footprint at 100 mM NaCl (Table 1)^12^,^44^. Furthermore, high coverage can additionally cause fluorescence self-quenching (Supplementary Fig. 8)^25^,^45^. These nonlinear effects complicate quantitative analyses of DNA intercalation and can be avoided by working at low dye coverage (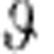
<<50%).

Our thorough characterization of DNA intercalation revealed that the kinetic rates can effectively be tuned by altering DNA tension, changing intercalating species and varying ionic strength. This valuable insight facilitates the selection of experimental conditions that range from a situation in which rapid kinetics cause DNA intercalation to always be in equilibrium, to a situation in which equilibration takes much longer than typical experimental timescales. Such experimental control over kinetic rates can be a powerful tool for investigations of the structural impact of intercalators in biomolecular studies. For example, conditions under which the intercalation kinetics are extremely slow (which yields a constant dye coverage even when stretching DNA) enabled us to systematically analyse how intercalators affect the mechanical properties of DNA. Intercalators are also known to affect DNA overstretching, which comprises a major structural transition of DNA^12^,^46^. We found that the interference of intercalators with DNA overstretching is much smaller when intercalator dissociation is fast than when it is slow. From these results it follows that the magnitude of the structural interference is largely governed by the dissociation rate, rather than by the absolute intercalator coverage or affinity alone. We, furthermore, showed that this useful insight can also be applied to DNA-processing (exonucleolysis) by replicative DNAPs. In particular, our data revealed that the use of intercalators with comparatively fast off-rates (for example, YO) minimizes the unwanted reduction in enzymatic activity that we observed for slower intercalators (for example, SbG and YOYO). Although the detailed response likely depends on the studied enzyme and conditions in question, we expect that our findings can have major implications for optimization of intercalator-based enzymatic activity assays, which are increasingly being exploited in, for example, (flow-)stretched DNA or DNA-curtain applications^5^,^6^,^7^.

The *in vitro* experiments performed here can additionally provide a deeper understanding of the *in vivo* impact of intercalators: an explicit relation between intercalation kinetics and the structural interference with DNA-processing enzymes is consistent with, for example, the extremely slow binding kinetics of the intercalating drug Actinomycin D^10^, a potent antibiotic and anticancer drug. An even more intricate role of the kinetics is known for the intercalating drug ditercalinium^22^, which dissociates slow enough to activate the DNA repair system, yet fast enough to repeatedly signal DNA repair and induce apoptosis. We expect that our approach can shed more light on the molecular mechanisms of the mutagenic and therapeutic effects of intercalating drugs *in vivo*, and that these findings can serve as a mechanistic basis for drug design.

## Methods

### DNA intercalators and buffers

Bacteriophage lambda (λ) DNA was biotinylated by filling in the 3′-recessive ends with biotinlyated nucleotides (biotin-14-dATP and biotin-14-dCTP) using the Exo-klenow fragment of DNAP I (New England Biolabs) and coupled to either 3.05 or 3.28 μm streptavidin-coated microspheres (Spherotech)^30^. SYTOX Orange, SYTOX Green, SYBR Gold, YO-PRO-1, YOYO-1 and POPO-3 were obtained from Invitrogen and stored at −20 °C in dimethylsulphoxide (DMSO). All data were obtained in a buffer of 20 mM Tris-HCl, pH 7.5 containing 0.01% Tween-20 complemented with NaCl at the concentration specified in the text. Buffers complemented with dye had DMSO concentrations below 2% v/v.

### Instrumentation

The experiments were performed on a custom-built inverted microscope that combines wide-field fluorescence microscopy and dual-trap optical tweezers^29^,^30^,^47^,^48^,^49^. In brief, we used a 1,064-nm fibre laser (YLR-10-LP, 10 W, IPG Photonics) and a water-immersion objective (Plan Apo × 60, numerical aperture 1.2, Nikon) to create two orthogonally polarized optical traps. The trap separation is controlled using a piezo mirror (Nano-MTA2X Aluminium, Mad City Labs) for beam-steering one trap. Force measurements are performed by back-focal plane interferometry^50^ of the condenser toplens (P 1.40 OIL S1 11551004, Leica) using a position-sensitive detector (DL100-7PCBA3, Pacific Silicon Sensor).

Fluorescence microscopy was achieved by imaging the stained DNA on an EMCCD camera (iXON+ 897E, Andor Technology). Here a 491-nm excitation laser (Cobolt Calypso 50 mW CW), and HQ540/80 m bandpass filter (Chroma Technology), was used for imaging of YO-PRO-1, YOYO-1, SYBR Gold and SYTOX Green, and a 532-nm excitation laser (Cobolt Samba 50 mW CW) and FF01–580/60 bandpass filter (Semrock Inc.) was used for imaging of SYTOX Orange and POPO-3.

A multichannel laminar flow cell (Micronit Microfluidics BV) was mounted on an automated XY-stage (MS-2000, Applied Scientific Instrumentation), which allowed rapid *in situ* construction and characterization of dumbbell constructs, and facilitated swift and complete transfer of the tethered DNA between different flow channels. The flow cell and microspheres are illuminated by a 450-nm light-emitting diode (Roithner Lasertechnik GmbH) and imaged in transmission on a CMOS camera (DCC1545M, Thorlabs).

A software package, custom-written in LabVIEW, was used to control optical trapping and microfluidics hardware. This software includes a force-feedback mode that adjusts the trap separation in order to maintain a constant tension on the DNA. Optical tweezers' data were analysed using LabVIEW and Origin. Fluorescence imaging was controlled using Micro-Manager, and images were analysed using ImageJ.

### Preparation of dye channel

Intercalators are well known to nonspecifically bind to glass surfaces and microfluidic tubing with high affinity (which is also mentioned by the supplier of the used cyanine dyes^51^,^52^)^25^,^31^. Such surface adsorption reduces the dye concentration in solution right after introducing dyes to the microfluidic system or after increasing the dye concentration. Surface desorption, on the other hand, increases the dye concentration when switching to a lower dye concentration. In order to ensure that during measurements a constant and well-defined dye concentration is present, it is crucial to equilibrate the microfluidic system with the dye-containing buffer. In equilibrium, surface adsorption equals dye desorption. Equilibration was accomplished by thorough flushing with a large volume of intercalator. We confirmed that the dye concentration was equilibrated by monitoring changes in DNA elongation. Here we applied a DNA tension that was high enough to ensure a significant lengthening signal, yet low enough to ensure that DNA intercalation occurs within an equilibration time less than 1 min. The channel was considered to be equilibrated in case the DNA elongation was unaltered after additional flushing and remained constant over repeated measurements. The channel equilibration time strongly depended on the intercalator used. For example, YOYO (which exhibits the strongest glass adsorption properties of all the dyes tested) required at least 24 h of flushing for equilibration.

### Determination of the DNA elongation per intercalator

We only performed these experiments for the most photostable dyes, SxO (mono-) and YOYO (bis-intercalator). The general idea behind the measurement was to use the fluorescence intensity signal to quantify the number of intercalators bound to the DNA, and simultaneously record the elongation with the optical tweezers. To this end, we first recorded the fluorescence intensities of single binding events using laser intensities and dye concentrations (<50 pM) that allowed single dye tracking (Supplementary Fig. 1). Next, we imaged the same DNA under conditions of higher dye coverage (>>1,000 dyes) to ensure sufficient DNA elongation (>500 nm) while not exceeding the limit in which self-quenching is relevant (<2.5 μm, compare Supplementary Fig. 8). Typical intercalator concentrations of 5 nM and higher were used. For these experiments, salt concentrations were used that ensured sufficiently long interaction times (>>1 s) at medium forces, but not too long such that the equilibration time was less than 10 min (50 mM NaCl for SxO and 500 mM NaCl for YOYO). For imaging, DNA tensions between 25 and 45 pN were applied. To minimize the impact of photobleaching, only the first frame of illumination was used to measure the brightness.

To determine the average brightness of a single dye, the intensity of a spot was determined in a region of interest (ROI) much larger than the spot diameter. The intensity of the event was then averaged over the duration of the event, excluding the first and last frames. Binding events closer than 1 μm to the beads were excluded, in analogy with the brightness determination on a strongly coated DNA (see below). The spot brightness was calculated for each event by subtracting the average background intensity in the same ROI either directly before or after the binding event. The average brightness of a binding event was then obtained as the centre of a Gaussian fit of the brightness histogram of >100 single dye-binding events (Supplementary Fig. 1c).

The brightness of a strongly coated DNA molecule was calculated analogous to the procedure described below; the background signal was measured by imaging two microspheres without a stretched DNA molecule. The number of bound dyes was then determined from the total brightness of a coated DNA divided by the average intensity of a single dye. Single Δ*x*-values were finally obtained from each measurement by dividing the deduced elongation (see below) by the corresponding number of bound dyes.

### Quantification of dye coverage through DNA elongation

DNA intercalation is quantified by measuring the difference in end-to-end length, Δ*L*, between bare DNA, *L*_0_(*F*), and intercalated DNA, *L*(*F*), where Δ*L* at a given coverage is assumed to be independent of applied tension (*cf.* Fig. 6a,b, equation (3) and Supplementary Fig. 4a,b). First, a DNA molecule, in absence of intercalators, is subjected to a force ramp in 6 pN steps from 6 to 60 pN (6–54 pN at 100 mM NaCl) in order to record the force-dependent end-to-end length of bare DNA, *L*_0_(*F*). Subsequently, the same DNA molecule was moved into the dye channel (the distance from the measurement location in the dye channel to the buffer channel was >2 cm such that the reduction in local dye concentration due to diffusion into the buffer channel was negligible over the timescale of the experiment). In the dye channel, the force ramp was repeated in presence of intercalators in order to obtain the force-dependent end-to-end length of intercalated DNA *L*(*F*). After each force step of the ramp, the change in DNA length due to intercalator binding was recorded until it had reached a constant level. Only in case the DNA–intercalator equilibration time exceeded 5 min, we stopped the measurement after reaching at least 90% of the equilibrium length change between force steps; the equilibrium length of the intercalated DNA was subsequently obtained by performing a single-exponential fit of the time-dependent elongation curve. For such long measurements, the dye solution was flushed through the channel between force steps in order to prevent reduced dye concentration due to diffusion into the buffer channel (during measurements, however, no flow was applied). Finally, the DNA elongation at a specific force, *F*, was defined as the difference between the end-to-end length of bare DNA and the equilibrium end-to-end extension of intercalated DNA, Δ*L*(*F*)=*L*(*F*)–*L*_0_(*F*).

### Quantification of dye coverage through fluorescence

We did not only quantify DNA intercalation by determining DNA elongation, but also using fluorescence microscopy. Fluorescence images (typical exposure time, 1 s) were only recorded for YO, SxO, SbG and YOYO, which are sufficiently photostable. Photobleaching was minimized by reducing excitation laser power such that fluorescence intensity differed at most 95% between subsequent images acquired at 60 pN (at which DNA–intercalator interaction time was longest). At elongations Δ*L*>1 μm (corresponding to roughly 1,000/500 dyes for mono/bis-intercalators), the signal-to-noise ratio of single fluorescence images were sufficient to reliably quantify the total fluorescence signal. At lower dye coverage, on the other hand, multiple images were averaged in order to compensate for the reduced signal-to-noise ratio. For example, three to five images were averaged to quantify the total fluorescence signal at Δ*L*∼100–200 nm, while up to ∼200 frames were averaged if only a few dyes per frame were present.

We calculated the fluorescence signal (a.u.) on the intercalated DNA from the (averaged) fluorescence images by summing the pixel values within a rectangular ROI centred on the DNA. The ROI was typically 1 μm wide in the direction perpendicular to DNA. In the direction parallel to the DNA, the ROI length corresponded to the DNA length reduced by a fixed value of 1 μm on both ends of the DNA in order to exclude artefacts from fluorescence that originated from the beads (autofluorescence and adsorbed dyes). The average intensity per unit length along the DNA was subsequently calculated by dividing the total ROI intensity by the ROI length and subtracting the average background intensity per unit length (which was obtained by an identical analysis of the two ROIs directly adjacent to first ROI in the direction perpendicular to the DNA). Finally, the total fluorescence signal due to DNA intercalation was calculated by multiplying the average intensity per unit length with the DNA extension obtained from the optical tweezers data.

### Fitting of thermodynamic and kinetic parameters

To extract force-dependent binding constants from our equilibrium DNA elongation data, we used the equation (derived from equations (1) and (2) and 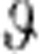
=Δ*L*/Δ*L*_max_, with Δ*L*_max_=Δ*x*_eq_*N*_bp_/*n*, see also Supplementary Note 1):


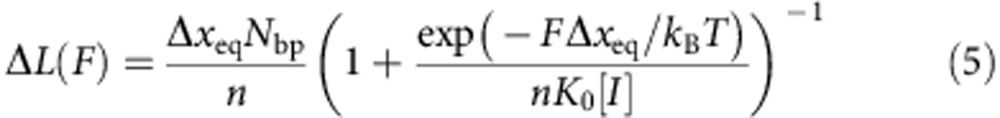


Here *N*_bp_ is the total number of basepairs in lambda DNA (*N*_bp_=48,502) and Δ*x*_eq_ is the DNA elongation per intercalator. *n*, *K*_0_ and Δ*x*_eq_ were used as fit parameters. Data sets of at least five DNA molecules were individually fitted, and the average of the fitted parameters was calculated. The error of the fitted parameters was estimated by the s.e.m. (*cf.* Table 1). Note that Δ*L*_max_ is not measured directly because of potential saturation effects (Supplementary Note 3). Instead, it follows implicitly from the fitting procedure.

### Direct measurement of dissociation rates

In order to directly analyse *k*_off_ for the DNA–intercalator interaction, we monitored the dye dissociation rate in the absence of free dye in solution. First, DNA was incubated in the dye channel under conditions that ensure a strong signal (elongation Δ*L*>1 μm). Subsequently, the intercalated DNA was moved into the buffer channel that lacks intercalator. At constant tension, dye dissociation results in a reduction of DNA length. The reduction of DNA length over time was then fitted with a single-exponential decay to yield the corresponding off-rate. For all intercalators, we observed increasing deviations from single-exponential decay (that is, an elevated off-rate) at increasing elongation (that is, at higher dye density; Δ*L*>0.5–1 μm) at 100 mM NaCl. With the exception of POPO, these deviations were no longer observed at 1,000 mM NaCl, even for elongations up to 4–5 μm (*cf.* Discussion section and Supplementary Fig. 8). In these cases, we restricted the fit to the single-exponential tail section of the decay (depending on the dye, Δ*L*<0.5–1 μm).

In all cases, we ensured that the DNA extension returned back to the original value of uncoated DNA, such that the DNA extension after the decay was always within a few percent of the elongation of the bare DNA (<50 nm). This can be deduced from Fig. 4, 5b,c, where the elongation is shown in many cases to drop below 0.01 μm. We demonstrate this more clearly in Supplementary Fig. 3a,b for two different dyes, SxO and POPO, by comparing the extension time course of the force clamp measurements with the stretching curve of bare DNA. When slow detachment (>10 min) made it impractical to follow the full relaxation (that is, the measured data did not decay to within 50 nm of the uncoated DNA), we checked complete dissociation by using the extension value extrapolated from a single-exponential fit of the decay. Finally, we never found any indication under the conditions tested, that dye-binding persisted on the DNA over longer timescales (>1 s) in another mode than intercalation; thus, when the DNA is restretched after de-intercalation, we did not find any substantial elongation or fluorescence increases that indicated re-intercalation of the dye (Supplementary Fig. 3c).

In principle, the response time of our force-feedback system permits the measurement of off-rates that correspond to 1/*k*_off_>0.1 s. In practice, however, contamination of the intercalator-free buffer due to dye diffusion sets boundaries to the off-rates that could be measured: in order to prevent such local contamination due to dye diffusion, we chose to move the DNA over a large distance of at least 2 cm away from the dye channel. Since such large movement takes ∼10 s, the fastest off-rates that we could reliably measure corresponded to 1/*k*_off_>10 s. Dye diffusion also limited the slowest off-rates that could be measured since measurement of off-rates that corresponded to 1/*k*_off_>1,000 s suffer from dye contamination due to diffusion over the required measurement time that exceeds ∼30 min.

An alternative method to obtain the off-rate without the need to exchange buffers is through measurement of the equilibration rate *k*_eq_ in presence of intercalators: using the relation *K*=*k*_on_/*k*_off_ and equation (1) and (9), the off-rate can be expressed in terms of *k*_eq_, and the equilibrium coverage, 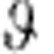, to yield:


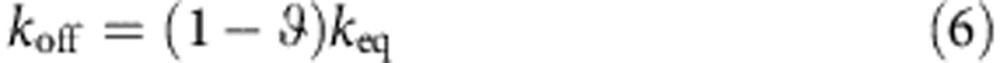


*k*_eq_ can be readily measured by monitoring the length equilibration (that is, the change in length over time at constant tension) that occurs after a sudden change in DNA tension in the presence of free intercalators. The length change over time can be fitted by a single exponential to obtain *k*_eq_, and 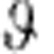 can be obtained from the corresponding equilibrium elongation at the same tension. This method, although less accurate than direct measurement of *k*_off_, is suitable for measuring off-rates that are too fast to allow for the 10-s buffer exchange (that is, YO at 100 mM and SxO at 1,000 mM NaCl).

A comparison of the method to measure *k*_off_ directly (that is, in absence of intercalators) with the method to measure *k*_off_ indirectly (that is, in presence of intercalators using equation (6)) shows that the two methods yield rates that are in good agreement with each other (*cf.* Fig. 5a).

### Force-extension curves at constant dye coverage

To analyse the mechanics of intercalated DNA at approximately constant coverage, we acquired force-extension curves within a time that is much shorter than the minimum DNA–intercalator equilibration time (that is, the time corresponding to 1/*k*_eq_ at the lowest tension). First, DNA was incubated in the dye channel to ensure sufficient elongation (Δ*L*>2 μm). The intercalated DNA was then moved to the buffer channel where repeated force-extension curves were acquired. Reliable force-extension curves (for which the DNA extension was increased over 12 μm in increments <40 nm) required at least 2 s. To ensure an approximately constant coverage over a single force-extension curve, we chose buffer conditions in which 1/*k*_eq_ at 0 pN exceeded 2 s. For YOYO, these conditions were satisfied by using a buffer of 20 mM Tris pH 7.5 and 25 mM NaCl. For SxG, we used 5 mM Tris and 10 mM NaCl, as well as fast stretching (>4 μm s^−1^). For both intercalators, we confirmed that these conditions yielded essentially constant dye coverages by checking that the difference in DNA elongation of two subsequent force-extension curves (acquired immediately after each other) was less than 5 %. Force-extension curves were acquired at different constant dye coverages by holding the DNA at 0 pN tension between two curves, in order to allow the intercalated dyes to dissociate (note that the lowest force corresponds to the shortest interaction time, *cf.* Fig. 4).

### Force-extension curves at equilibrium dye coverage

To obtain force-extension curves under equilibrium intercalator coverages, the curve acquisition time needs to greatly exceed the DNA–intercalator equilibration time. First, we acquired a force-extension curve of bare DNA in an intercalator-free buffer. Subsequently, the DNA was moved to the dye channel where a force-extension curve was acquired in presence of intercalators under equilibrium conditions. For the bis-intercalator POPO, these conditions are satisfied at 20 mM Tris pH 7.5 and 1,000 mM NaCl and a slow stretching rate (<25 nm s^−1^). For the mono-intercalator YO, we used 1,000 mM NaCl and a stretching rate of 1 μm s^−1^. Equilibrium conditions were confirmed by checking that the hysteresis between force-extension curves recorded in forward and backward directions was negligible. Equation (4) was used to fit the equilibrium force-extension curves. Here the experimental force-extension curve of bare DNA was used as *L*_0_(*F*), while *K*_0_, *n* and *Φ*_eq_ were used as fit parameters. Alternatively, one could use polymer mechanics models such as the worm-like chain model for *L*_0_(*F*).

### DNAP activity

T7 DNAP was acquired from New England Biolabs. In order to analyse the DNAP exonuclease activity, we employed buffer conditions as specified by the supplier, under which the DNAP activity is highly processive (that is, interaction time >10 s; the exonucleolysis buffer consisted of 20 units ml^−1^ polymerase DNAP,10 mM Tris-HCl pH 7.5, 50 mM NaCl, 2 mM MgCl_2_, 0.02 % BSA and 0.01% Tween-20). λ-DNA was modified to contain a single 3′-recessive end at which DNAP activity can initiate^37^. Experiments were started by moving DNA into a channel containing DNAP. DNAP activity was studied by monitoring the change in DNA extension at a constant tension of 40 pN, which ensures a high exonuclease activity. The change in DNA extension, acquired at a sampling rate of 20 Hz, was used to calculate the amount of digested dsDNA. For this, the recorded DNA extension increase/decrease (Δ*L*^DNAP^) was converted to the corresponding amount of dsDNA digested/polymerized by DNAP activity 
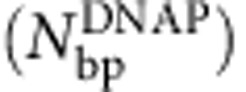
. As described previously, this was accomplished using the formula 
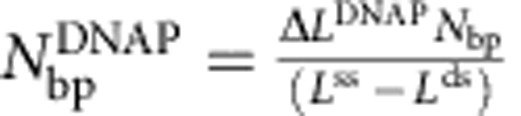
, where *L*^ss/ds^ denotes the end-to-end length of fully single- and dsDNA, respectively, at a tension of 40 pN (ref. 36).

To create histograms of the average exonucleolysis rate, a linear fit of the amount of digested DNA as function of time was performed within a sliding window of 100 points or 5 s. Mono-intercalators were added to study their effect on DNAP activity. The intercalator concentrations were chosen such that the elongation of the lambda DNA before exonucleolysis was ∼Δ*L*=250–300 nm at 40 pN (corresponding to ∼800 dyes over the length of the full DNA).

In order to analyse the polymerization activity of DNAP, we employed a polymerization buffer containing 10 mM Tris-HCl pH 7.5, 50 mM NaCl, 4 mM MgCl_2_, 2 mM dNTP and 0.01% Tween-20, complemented with 40 units ml^−1^ DNAP. First, high tension (45–55 pN) was applied in order to induce processive exonuclease activity and digest 5–10 kbp dsDNA. A long section of ssDNA is thus created that serves as a template for polymerization activity. Next, the tension was decreased to 15 pN, at which the predominant DNAP activity is polymerization rather than exonucleolysis. The length changes were converted to the amount of single-stranded bases and the average polymerization rate was calculated as described above. For analysing the polymerization activity in presence of intercalator, two enzyme solutions were used. First, a long ssDNA section was created through exonucleolysis in the exonucleolysis buffer described above. No intercalators were added to avoid reduced exonucleolysis activity. Next, the DNA was moved to another channel that contained the polymerase buffer complemented with YO. We employed a YO concentration that resulted in a dye coverage similar to the dye coverage used in the exonucleolysis experiments.

## Additional information

**How to cite this article:** Biebricher, A. S. *et al.* The impact of DNA intercalators on DNA and DNA-processing enzymes elucidated through force-dependent binding kinetics. *Nat. Commun.* 6:7304 doi: 10.1038/ncomms8304 (2015).

## Supplementary Material

Supplementary Information Supplementary Figures 1-8, Supplementary Table 1, Supplementary Notes 1-3 and Supplementary References

## Figures and Tables

**Table 1:** Measured thermodynamic and kinetic parameters of DNA-intercalating cyanine dyes.

**Dye**	**[NaCl] (M)**	**Equilibrium**				Dissociation*			Association*		
		***K***_**0**_ **(M**^−1^)	***n*** **(bp)**	**Δ*x***_**eq**_ **(nm)**	***Φ***_**eq**_ **(pN)**	***k***_**off,0**_^−1^ **(s)**	**Δ*x***_**off**_ **(nm)**	***Φ***_**off**_ **(pN)**	***k***_**on,0**_ **(M**^−1^**s**^−1^)	**Δ*x***_**on**_ **(nm)**	***Φ***_**on**_ **(pN)**
**YO**	0.1	(2.9±0.9) × 10^5^	3.8±1.0	0.31±0.03	13.1	0.009±0.004	0.28±0.04	14.6	(3.2±1.7) × 10^7^	0.03±0.05	151
	1.0	(3.4±0.9) × 10^3^	2.1±0.6	0.35±0.03	11.7	†	†	†	†	†	†
**SxO**	0.1	(2.4±0.5) × 10^6^	3.0±0.4	0.30±0.02	13.5	0.31±0.08	0.24±0.04	17.4	(7.9±2.6) × 10^6^	0.07±0.04	63
	1.0	(4.4±0.7) × 10^4^	2.4±0.6	0.32±0.01	13.0	0.23±0.02	0.14±0.02	28.4	(2.0±0.3) × 10^5^	0.18±0.02	23
**SbG**	0.1	(7.8±3.3) × 10^6^	3.2±0.5	0.30±0.01	13.9	0.61±0.17	0.28±0.03	14.5	(1.2±0.6) × 10^7^	0.02±0.03	173
	1.0	(4.5±1.2) × 10^5^	2.1±0.5	0.35±0.03	11.9	0.21±0.05	0.23±0.04	17.9	(2.1±0.8) × 10^6^	0.12±0.05	33
**SxG**	0.1	(1.4±0.3) × 10^7^	2.6±0.6	0.27±0.02	15.5	0.51±0.24	0.31±0.05	13.2	(2.6±1.1) × 10^7^	−0.04±0.05	−118
	1.0	‡	‡	‡	‡	‡	‡	‡	‡	‡	‡
**YOYO**	0.1	§	§	§		48±17	0.61±0.10	6.8	§	§	§
	1.0	(2.8±1.4) × 10^4^	4.7±0.7	0.71±0.04	5.8	0.09±0.06	0.58±0.07	7.1	(3.3±2.8) × 10^5^	0.13±0.08	31
**POPO**	0.1	(8.5±6.0) × 10^4^	6.5±2.3	0.55±0.07	7.5	0.10±0.06	0.56±0.11	7.3	(6.1±5.7) × 10^5^	0.02±0.16	230
	1.0	(9.5±6.6) × 10^2^	4.8±0.7	0.67±0.06	6.1	0.05±0.02	0.46±0.26||	8.8||	(1.2±1.0) × 10^4^	0.29±0.27||	14.1||

^*^Δ*x*_off/on_=*k*_B_*T*/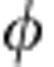
_off/on_.

^†^*k*_off,0_ is too fast to be measured (*k*_off,0_^−1^<<0.01 s).

^‡^*K*_0_ is too low to be measured.

^§^Equilibration is too slow to measure *K*_0_.

^||^Because *k*_off_ exhibited a nonexponential force dependence at high force, data fitting was restricted to 0<*F*≤24 pN (Fig. 5h).
