# Supplementary material for: The impact of DNA intercalators on DNA and DNA-processing enzymes elucidated through force-dependent binding kinetics
Source: Nat Commun. 2015 Jun 18;6:7304. doi: 10.1038/ncomms8304 (PMC4557362; doi:10.1038/ncomms8304)
Supplement: Supplementary Information — Supplementary Figures 1-8, Supplementary Table 1, Supplementary Notes 1-3 and Supplementary References [file ncomms8304-s1.pdf]

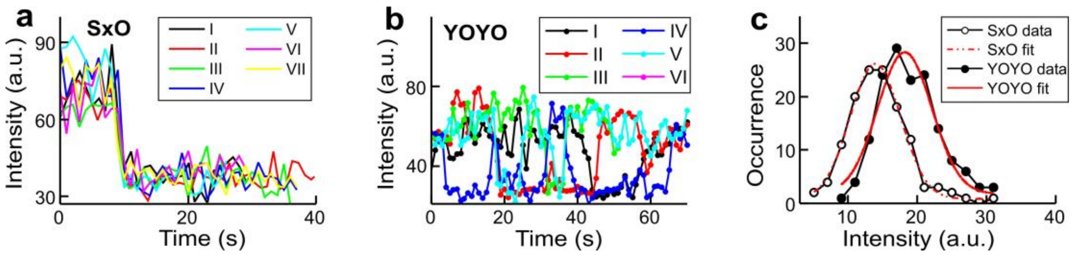

Supplementary Figure 1. Discrete binding events at low intercalator concentration display single fluorophore characteristics. (**a**) The fluorescence intensity of seven examples of SxO binding events shows photobleaching in a single step to background intensity. For clarity, traces have been coaligned along the time-axis. (**b**) Under low salt conditions (50 mM NaCl), six examples of YOYO binding events display intermittent fluorescence emission (blinking), i.e., the intensity drops to background level but recovers after some time back to the original level. (**c**) Brightness histograms of discrete binding events both for SxO in 50 mM (open) and YOYO in 500 mM NaCl (closed circles) follow a homogenous distribution and can be well fitted with a Gaussian curve (red). The procedure for measuring the brightness is described in **Methods**.

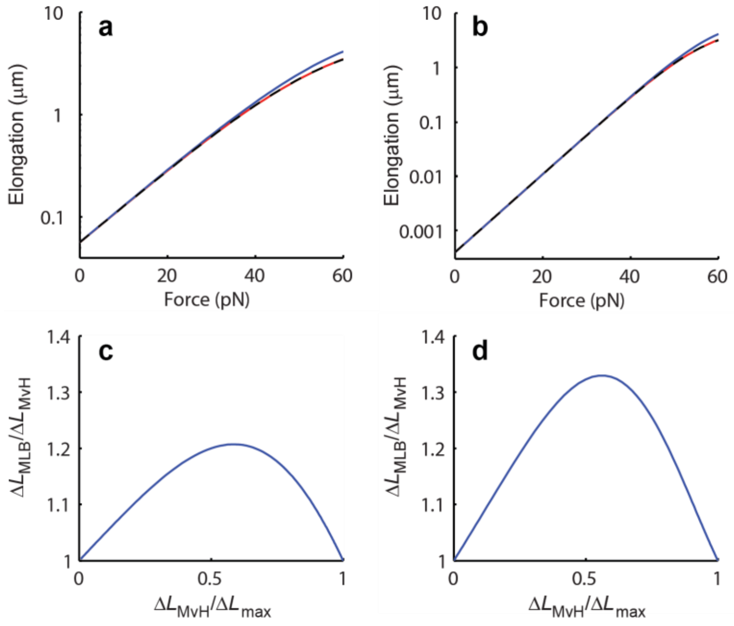

Supplementary Figure 2. Comparison of DNA elongation calculated using the multi-ligand binding and McGhee-von Hippel binding isotherms for a mono-intercalator (**a**,**c**), and a bis-intercalator (**b**,**d**) (see **Supplementary Note 1**). (**a**,**b**) DNA elongation calculated using multi-ligand binding isotherm (blue), and McGhee-von Hippel binding isotherm (red). Parameters were chosen such that the dye coverage at 60 pN is about 50% of saturated coverage. The dashed black line indicates a fit of equation (5) (DNA elongation according to multi-ligand binding) to the DNA elongation calculated using McGhee-von Hippel. (**c**,**d**) The ratio of the DNA elongation calculated using multi-ligand binding (ΔL_{MLB}) and the DNA elongation calculated using McGhee-von Hippel (ΔL_{MvH}), as function of the relative coverage $\Delta L_{\mathrm{MvH}}/\Delta L_{\mathrm{max}}$.

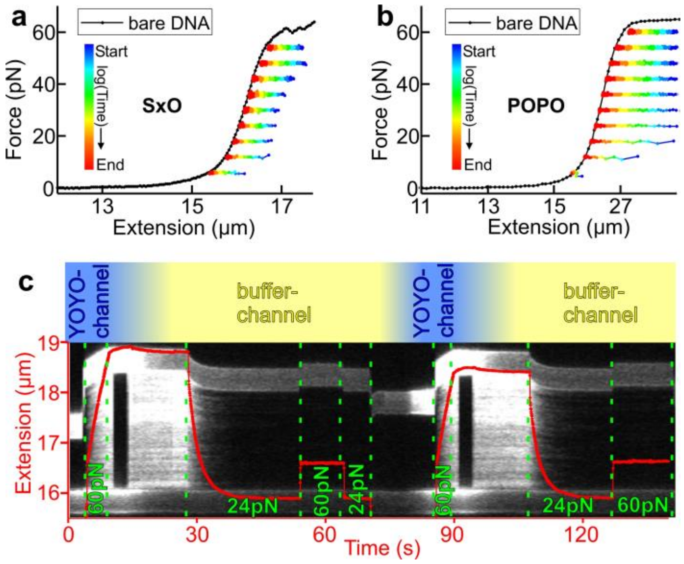

Supplementary Figure 3. Confirmation that during de-intercalation experiments the dyes dissociate completely from the DNA. (**a,b**) Time-traces of the extension decrease due to to dye de-intercalation (coloured) at different tensions (6-60 pN) result in complete return to the bare DNA length for SxO at 100 mM (**a**) and POPO at 1000 mM NaCl (**b**). De-intercalation measurements start (blue) at extensions more than 1 µm longer than the uncoated DNA (black) and display complete relaxation of DNA to its length before dye incubation at the end of the measurement (red). In cases where the off-rate is too slow to follow complete equilibration on experimentally practicable timescales (e.g., traces at 48/54 pN in **3a**, and at 60 pN in **3b**), we used single-exponential fits to extract the extension in equilibrium and confirmed that this extrapolated length corresponds within error to the bare DNA length.

(**c**) De-intercalation reflects complete dye unbinding for YOYO. The experiments starts in the YOYO channel (blue shading) at very low force where virtually no binding occurs due to the low affinity. After a few seconds the force clamp (green vertical dashed line) is activated at 60 pN, causing rapid intercalation due to the strongly increased affinity, as can be observed from the increase of fluorescence intensity (kymograph) and DNA extension (red curve). As a result of the low off-rate at high tension, the dye stays bound when the force clamp is switched off, and the construct is moved (at ~10 s) out of the dye- (blue) into the buffer-channel (yellow shading). After clamping the force again, now at 24 pN (at ~28 s), the strongly increased off-rate results in fast de-intercalation (evident from the decrease in extension and the drop in fluorescence intensity). When the force is increased, by clamping again at 60 pN, neither extension nor fluorescence intensity show a significant increase. This latter observation shows unequivocally that the intercalator has completely detached from the DNA and diffused away, and therefore cannot re-intercalate. After moving the construct back into the YOYO channel (at ~80 s), the whole circle of binding and unbinding can be re-initiated, demonstrating that the dye intercalation properties of the DNA have not changed.

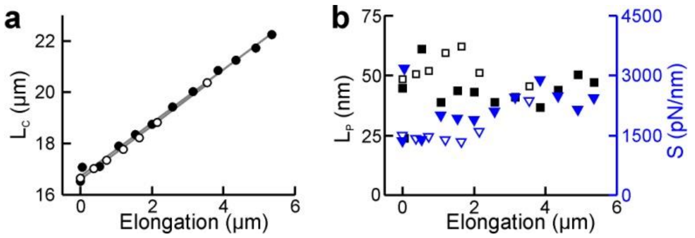

Supplementary Figure 4 Effect of intercalation on DNA mechanics: Intercalation does not affect stretch parameters other than the contour length for both mono- (SxG, open) and bis-intercalators (YOYO, closed symbols). Force-extension curves obtained under constant dye coverage (cf. **Fig 6**) were analyzed using the extensible worm-like chain model.[1] (**a**) The fitted contour lengths, L_c, display a linear dependence on the intercalator-induced DNA elongation, with a slope close to 1: The linear fits (gray) indicate slopes of 1.05 and 1.03 for SxG and YOYO, respectively, which confirms that DNA elongation is an accurate measure for quantifying DNA intercalation. (**b**) The fitted persistence length (L_p, black squares) and stretch modulus (S, blue triangles) exhibit no clear correlation with the DNA elongation.

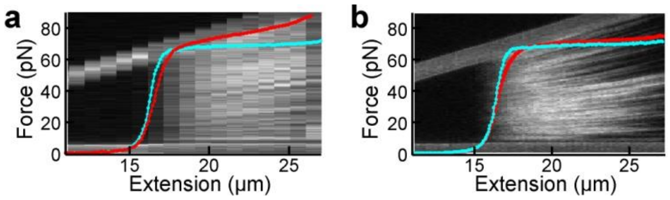

Supplementary Figure 5. The effect of intercalation on DNA overstretching depends on the DNA-intercalator binding kinetics. Both panels show force-extension data obtained in the presence of SbG at 1000 mM NaCl concentration (red curves), and the corresponding kymographs obtained from fluorescence images. Cyan curve, force-distance data of bare DNA. Fast stretching (**a**, ~2.5 µm/s) results in a homogeneous stretch pattern (kymograph) accompanied with an inclined force 'plateau' during overstretching, which indicates that the intercalator perturbs the cooperative DNA overstretching transition (cf. **Fig. 7a,b**). Slow DNA stretching under identical conditions (same dye coverage at 60 pN), on the other hand (**b,** ~0.5 µm/s), yields a kymograph that displays inhomogeous darkening along the DNA as well as a smaller force increase in the overstretching region, which resembles our previous observations that led to the conclusion that intercalators with fast dissociation rates exhibited minimal perturbation (**Fig. 7c**). These results demonstrate that minimal perturbation occurs if the stretching rate is slow enough in comparison to the dye detachment rate (0.2 s^{-1} at 60 pN), such that cooperative DNA overstretching can take place.

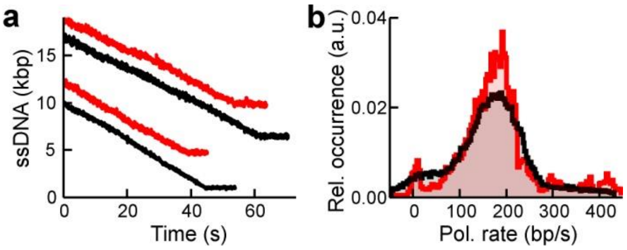

Supplementary Figure 6. Intercalator binding does not have a significant effect on T7 DNA polymerase activity. (**a**) Two typical polymerization traces acquired in the absence (black) and presence of YO (red, ~800 dyes/full DNA) show similar polymerase activity (as characterized by the reduction in the number of ssDNA bases at 15 pN). (**b**) Histograms of the average DNA polymerase rate show that intercalators do not reduce the average polymerization rate (as is expected since DNA polymerization proceeds on a ssDNA template, to which intercalators do not bind).

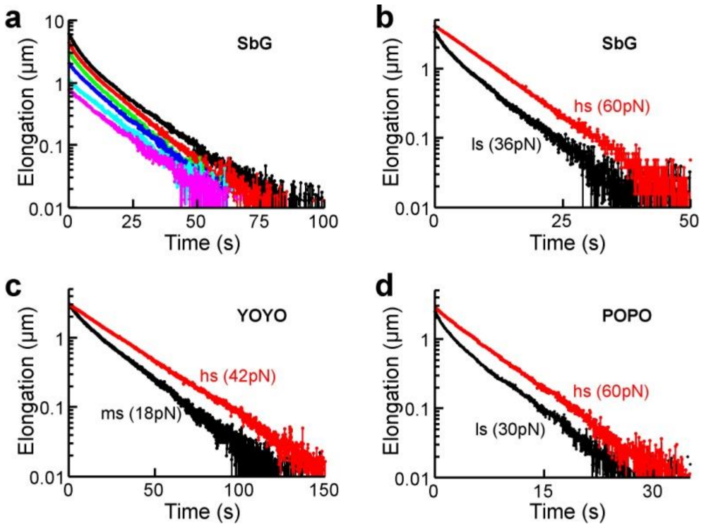

Supplementary Figure 7: Deviation from single-exponential dissociation dynamics; electrostatic repulsion of adjacent dyes at high dye coverage and low ionic strength results in an elevated off-rate. (**a**) Direct measurement of the dissociation dynamics of SbG-intercalated DNA in an intercalator-free buffer (100 mM NaCl) shows that SbG dissociation exhibits a time-dependence that deviates from single-exponential behavior (different colors indicate dissociation curves acquired for varying values of the initial dye coverage). More specifically, at elongations exceeding 1 µm the dissociation rate is elevated (i.e., the interaction time is reduced). A single-exponential 'tail' with an unaltered decay rate can however be observed for all curves in the low dye coverage regime. These observations can be explained by destabilization of the DNA-intercalator interaction if the dyes are in close proximity, resulting in an elevated off-rate until the residual dye coverage is low enough such that dye-dye repulsion is negligible. (**b-d**) Example traces of dye de-intercalation time courses for SbG (**b**), YOYO (**c**) and POPO (**d**) in different salt conditions; for comparison, the force-clamp tension (indicated in brackets) is chosen to give at different salt concentrations similar decay rates. In all cases, NaCl concentrations of 100-300 mM yielded strong deviations from single-exponential decay at higher dye coverages (black). The fact that no deviation from single-exponential decay is observed under conditions of stronger electrostatic screening (1000 mM NaCl, red) indicates that electrostatic repulsion of the positively charged dyes most likely causes the elevated off-rate.

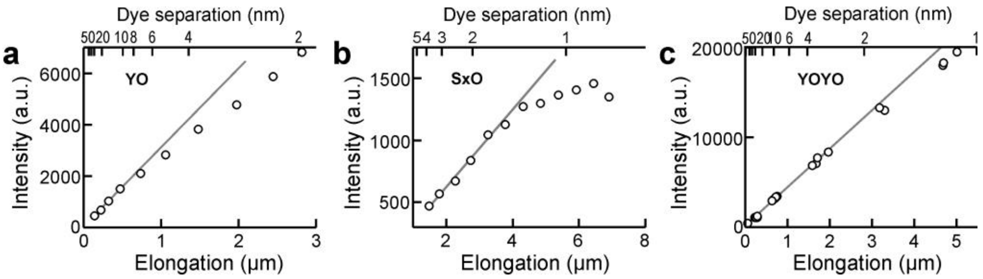

Supplementary Figure 8. Self-quenching of intercalator fluorescence due to close proximity of dyes at elevated coverages. Fluorescence intensity versus elongation and average dye separation for YO (**a**), SxO (**b**) and YOYO (**c**) shows a sub-linear increase in fluorescence with increased dye coverage (i.e. increased elongation). Gray lines indicate a linear dependence. This data indicates that the chromophoric systems of the dyes can couple in case dyes are in close proximity (on average 1-10 nm, depending on the dye), which can lead to non-radiative decay processes that preclude fluorescent decay (self-quenching), as has been reported previously.[2]

Supplementary Table 1 : Data-analysis based on the Multi-Ligand and McGhee-von Hippel binding isotherms

		Multi-Ligand Binding			McGhee-von Hippel		
Dye	**[NaCl] (M)**	K_0 (M^{-1})	Δx_{eq} (nm)	n (bp)	K_0 (M^{-1})	Δx_{eq} (nm)	n (bp)
**YO**	0.1	2.9×10^5	0.31	3.8	3.0×10^5	0.31	2.5
	1.0	3.4×10^3	0.35	2.1	3.4×10^3	0.35	1.7
**SxO**	0.1	2.4×10^6	0.30	3.0	2.6×10^6	0.30	2.3
	1.0	4.4×10^4	0.32	2.4	4.5×10^4	0.31	1.8
**SbG**	0.1	7.8×10^6	0.30	3.2	7.8×10^6	0.30	2.3
	1.0	4.5×10^5	0.35	2.1	4.4×10^5	0.35	1.7
**SxG**	0.1	1.4×10^7	0.27	2.6	1.4×10^7	0.27	2.1
**YOYO**	1.0	2.8×10^4	0.71	4.7	2.8×10^4	0.71	3.3
**POPO**	0.1	8.5×10^4	0.55	6.5	9.5×10^4	0.55	4.5
	1.0	9.5×10^2	0.67	4.8	6.3×10^2	0.68	3.4

Supplementary Note 1: Derivation of equations used for binding equilibrium and kinetics

In order to analyze the DNA-binding dynamics and equilibria of intercalating ligands, we set out to derive simple expressions for the average number of bound ligands per basepair, $\gamma(t)$, assuming an effective footprint per bound ligand of n basepairs. In the low coverage regime, every basepair (rather than every n^{th} basepair) is a potential binding site (here, we neglect neighbour exclusion effects that reduce the number of potential binding sites at higher coverage). The instantaneous binding and unbinding rates are thus given by, respectively, $r_{\mathrm{on}} = (1 - n\gamma)[I]k_{\mathrm{on}}$ and $r_{\mathrm{off}} = \gamma k_{\mathrm{off}}$, where k_{on} and k_{off} are the effective on-rate and off-rate. The rate of change of $\gamma(t)$ is thus:

$$\frac{\delta\gamma}{\delta t} = (1 - n\gamma)[I]k_{\mathrm{on}} - \gamma k_{\mathrm{off}} \qquad (7)$$

Under the simplifying assumption that k_{on} and k_{off} are independent of γ, equation (7) is a first-order linear differential equation that can be solved to describe the equilibration dynamics:

$$\gamma(t) = \gamma_{\mathrm{eq}} + (\gamma_0 - \gamma_{\mathrm{eq}})e^{-k_{\mathrm{eq}}t} \qquad (8)$$

Here, γ_0 represents the number of bound ligands per basepair at t = 0, and the equilibration rate, k_{eq}, is given by:

$$k_{\mathrm{eq}} = n[I]k_{\mathrm{on}} + k_{\mathrm{off}} \qquad (9)$$

The number of ligands per basepair in equilibrium is given by:

$$\gamma_{\mathrm{eq}} = \frac{1}{n + \frac{k_{\mathrm{off}}}{[I]k_{\mathrm{on}}}} \qquad (10)$$

By rewriting the latter expression using $K = k_{\mathrm{on}}/k_{\mathrm{off}}$ and $\vartheta = n\gamma_{\mathrm{eq}}$, we arrive at the multi-ligand binding isotherm that was described previously by equation (1).

Note that the multi-ligand binding isotherm is expected to deviate from DNA-binding data at high ligand coverage where many free binding sites are expected to be occluded because they are smaller than the ligand's footprint. The (non-cooperative) McGhee-von Hippel binding isotherm, on the other hand, does take these effects into account:[3]

$$\gamma = K[I]\frac{(1-n\gamma)^n}{(1-n\gamma+\gamma)^{n-1}} \qquad (11)$$

A direct comparison of the two binding isotherms through numerical calculation of the DNA elongation due to bound intercalators shows that the multi-ligand binding isotherm, equation (10), slightly overestimates the dye coverage at high binding density compared to the McGhee-von Hippel isotherm, equation (11) (**Supplementary Fig. 2a,b**). This overestimation is largest when the dye coverage is at about 50 % of its maximum value at complete saturation (~20% deviation for mono-intercalators and ~33% deviation for bis-intercalators, cf. **Supplementary Fig. 2c,d**). To quantify the expected effect of this overestimation on our data-analysis, we fitted equation (5) (DNA elongation based on multi-ligand binding, **Methods**) to the DNA elongation that we calculated based on the McGhee-von Hippel isotherm (**Supplementary Fig. 2a,b**).The fitted parameters for K_0 and Φ agreed within 0.4% and 2%, respectively. n, however, deviated substantially (36% and 59% for mono-and bis-intercalators, respectively), because its value is mostly determined by the behavior near saturation, where the two isotherms exhibit the largest difference. Indeed, a similar trend is observed if we compare an analysis of our experimental data using the multi-ligand binding isotherm (cf. **Table 1**) to an analysis using the McGhee-von Hippel binding isotherm (cf. **Supplementary Table**): both K_0 and Φ are in excellent agreement and n deviates by up to 52 % (31% on average). It is interesting to note, however, that at high ionic strength (i.e. at 1000 mM NaCl, where electrostatic repulsion has minimal effect on the apparent footprint, see Discussion section), data analysis using the multi-ligand binding isotherm yields an apparent footprint that only slightly exceeds the expected value of 2 basepairs per intercalating moiety.[4] The McGhee-von Hippel isotherm, on the

other hand, yields an apparent footprint that is slightly smaller than the expected value. Similar apparent shortcomings of the McGhee-von Hippel isotherm have been highlighted previously.[5] Since the apparent footprint n is additionally affected by electrostatic dye-dye repulsion,[6] we conclude that for both of the isotherms used, the estimated value of n should be interpreted with caution. K_0 and Φ (i.e. Δx), on the other hand, are accurately estimated by both isotherms. We therefore chose to use the simplest binding isotherm (multi-ligand binding) for the analysis of our data, which was mostly acquired at dye coverages well below saturation.

Supplementary Note 2: The studied cyanine intercalators predominantly affect DNA structure by increasing the contour length only

In **Fig. 6a,b** we show direct stretching data of the slow intercalators SxG and YOYO under constant dye coverage that demonstrate that these dyes lengthen the DNA and do not significantly affect persistence length or stretch modulus (**Supplementary Fig. 4**). This provides the justification for the general use of elongation as a linear measure for intercalated dye coverage and the application of equations (3) (4), and (5).

DNA cannot be stretched under constant coverage such as shown in **Fig. 6a,b**, when intercalators (un)bind fast, thus for these cases a validation cannot be achieved in a direct manner. Nevertheless, our data provide strong, indirect evidence that the elongation scales linearly with the dye coverage also in these cases. For SbG, SxO, YO we demonstrate (**Fig. 2a**, **Supplementary Fig 2a-e**) that fluorescence increases linearly with DNA elongation. The fluorescence intensity is a direct measure of the dye coverage (at relatively low coverage where fluorescence self-quenching does not occur (**Supplementary Fig. 8)**). Combined with the fact that this data was determined at different DNA tensions, we can show that indeed the elongation is independent of the DNA tension, which is the basis of equations (3)-(5). These observations furthermore indicate that intercalation by SbG SxO and YO only affects DNA contour length and not persistence length or stretch modulus, as shown for SxG and YOYO in **Fig. 6a,b**.

This leaves POPO as the only intercalator studied for which we cannot provide experimental evidence for it only affecting DNA contour length and not persistence length or stretch modulus. The chemical structure of POPO is, however, very similar to TOTO and YOYO. For these latter two intercalators, the structure of the bis-intercalation complex has been determined,[7,8] showing no indications of significant distortion of the DNA structure (e.g. kinking) that would result in a change of the persistence length. The chemical difference between TOTO, YOYO and POPO lies solely in the planar aromatic system that is responsible for the base-stacking properties of the dyes. This comparison of the structures of these related intercalators provides a reasonable basis for the assumption that POPO, just like its close relatives, mainly lengthens DNA upon intercalation and does not affect persistence length or stretch modulus.

Supplementary Note 3: Determination of the values Δx_{eq} and n

The term $\Delta x_{\mathrm{eq}} N_{\mathrm{bp}}/n$ in equation (5) has unit length and gives the DNA elongation due to intercalation at complete coverage,; for this reason it is also referred to as the maximum elongation ΔL_{max}. In principle, this parameter could be measured directly by measuring the elongation under saturating conditions. In the specific case of cyanine intercalators, however, we instead prefer to deduce this value indirectly by fitting Δx_{eq} and n in equation (5), for the following reasons. (I) In several cases (in particular at high salt), the intercalator concentrations required to achieve saturation is not accessible with commercially available stock concentration without surpassing 2% v/v DMSO in the final dilution. (II) As we demonstrate below, near saturation, effects such as dye-dye repulsion[9] (see **Supplementary Fig. 7**) and self quenching[2,10] (**Supplementary Fig. 8**) can affect the (apparent) dye coverage, and thus yield different results (e.g. regarding the footprint) than in the low coverage regime. (III) In addition, secondary binding modes distinct from intercalation have been demonstrated for cyanine dyes at high coverage,[10-15] making it impossible to consider this high-coverage regime without explicitly taking into account these additional binding modes. Instead, we determined Δx_{eq}- and n-values from fits (equation 5). The high dynamic range of our unique combined fluorescence optical tweezers approach, together with the high quality of the fits, provides solid support that our approach results in a good estimation of both Δx_{eq}- and n-values that are valid in the low coverage regime.

SUPPLEMENTARY REFERENCES

1. Odijk, T. Stiff Chains and Filaments under Tension. *Macromolecules* **28,** 7016–7018 (1995).

2. Yan, X. *et al.* Probing the kinetics of SYTOX Orange stain binding to double-stranded DNA with implications for DNA analysis. *Anal. Chem.* **77,** 3554–62 (2005).

3. McGhee, J. D. & von Hippel, P. H. Theoretical aspects of DNA-protein interactions: co-operative and non-co-operative binding of large ligands to a one-dimensional homogeneous lattice. *J. Mol. Biol.* **86,** 469–89 (1974).

4. Ihmels, H. & Otto, D. in *Supermolecular Dye Chem.* (Würthner, F.) **258,** 161–204 (Springer Berlin Heidelberg, 2005).

5. Van der Heijden, T. & Dekker, C. Monte carlo simulations of protein assembly, disassembly, and linear motion on DNA. *Biophys. J.* **95,** 4560–9 (2008).

6. Vladescu, I. D., McCauley, M. J., Nuñez, M. E., Rouzina, I. & Williams, M. C. Quantifying force-dependent and zero-force DNA intercalation by single-molecule stretching. *Nat. Methods* **4,** 517–22 (2007).

7. Spielmann, H. P.; Wemmer, D. E.; Jacobsen & J. P. Solution structure of a DNA complex with the fluorescent bis intercalator TOTO determined by NMR spectroscopy *Biochemistry* **34**, 8542-53 (1995).

8. Johansen, F., Jacobsen, J. P. 1H NMR studies of the bis-intercalation of a homodimeric oxazole yellow dye in DNA oligonucleotides. *J. Biomol. Struct. Dyn.* **16**, 205-22 (1998).

9. Wilson, W. D. & Lopp, I. G. Analysis of cooperativity and ion effects in the interaction of quinacrine with DNA. *Biopolymers* **18,** 3025–41 (1979).

10. Larsson, A., Carlsson, C., Jonsson, M. & Albinsson, B. Characterization of the Binding of the Fluorescent Dyes YO and YOYO to DNA by Polarized Light Spectroscopy. *J. Am. Chem. Soc.* **116,** 8459–8465 (1994).

11. Petty, J. T., Bordelon, J. A. & Robertson, M. E. Thermodynamic Characterization of the Association of Cyanine Dyes with DNA. *J. Phys. Chem. B* **104,** 7221–7227 (2000).

12. Yan, X., Habbersett, R. C., Cordek, J. M., Nolan, J. P., Yoshida, T. M., Jett, J. H. & Marrone, B. L. Development of a mechanism-based, DNA staining protocol using SYTOX Orange nucleic acid stain and DNA fragment sizing flow cytometry. *Anal. Biochem.* **286**, 138–148 (2000).

13. Cosa, G., Focsaneanu, K. S., McLean, J. R., McNamee, J. P. & Scaiano, J. C. Photophysical properties of fluorescent DNA-dyes bound to single- and double-stranded DNA in aqueous buffered solution. *Photochem. Photobiol.* **73**, 585–599 (2001).

14. Karlsson, H. J., Lincoln, P. & Westman, G. Synthesis and DNA binding studies of a new asymmetric cyanine dye binding in the minor groove of [poly(dA–dT)](2). *Bioorg. Med. Chem.* **11**, 1035–1040 (2003).

15. Zipper, H., Brunner, H., Bernhagen, J.; Vitzthum, F. Investigations on DNA intercalation and surface binding by SYBR Green I, its structure determination and methodological implication. *Nucleic Acids Res.* **32**, e103 (2004).
